# A single brief cue leaves a day-long internal state imprint in planarians

**DOI:** 10.1073/pnas.2606749123

**Published:** 2026-06-16

**Authors:** Ojeiru Felix Ezomo, Rena Suzuki, Maria Narahashi, Satoshi Matsuo, Takeshi Inoue

**Affiliations:** ^a^ Division of Adaptation Physiology, Faculty of Medicine, Tottori University, Yonago, Tottori 683-8503, Japan

**Keywords:** internal states, nonassociative plasticity, temporal integration, state transition, planarians

## Abstract

Behavioral decisions are elicited by environmental stimuli, but they are critically modulated by internal states. Persistent state-dependent biases are typically attributed to accumulated sensory history, high-intensity stimulation, or sustained environmental conditions. However, organisms also encounter isolated, innocuous perturbations whose long-term impacts remain poorly understood. Here, we show that a single brief mechanical cue induces changes in spontaneous locomotor activity in the planarian *Dugesia japonica* that persist over a day-long timescale. This cue specifically shortened rest bouts without altering within-bout movement kinematics, indicating a targeted shift in the transition probability out of rest rather than a general upregulation of motor activity. Furthermore, as this day-scale effect decayed, an underlying ultradian alternation in activity persisted. Our results demonstrate that postcue behavioral organization adopts a two-timescale structure, combining day-scale persistence with multihour patterning. This dual-layer architecture provides an empirical basis for understanding how a single environmental encounter can reprogram long-term behavioral patterns without further sensory input, extending the temporal reach of even compact nervous systems.

Animal behavior is modulated by internal states such as metabolic status, emotion, and arousal, rather than being merely reflex-like responses to immediate sensory inputs (1–3). These states serve as internal readouts of an animal’s physiological condition, biasing how incoming sensory information guides adaptive action selection. Such states can outlast the inducing stimulus and provide sustained behavioral modulation. Long-lasting behavioral shifts are typically induced by sustained environmental changes, repetitive training, or high-intensity experiences (4). However, it remains unclear how a single, minor transient event updates these internal states to dictate the temporal dynamics of the resulting behavioral bias.

Simple nervous systems offer experimentally tractable models for addressing these questions. The freshwater planarian *Dugesia japonica* is an early-branching bilaterian with a structurally compact brain (5) that displays a rich repertoire of spontaneous behaviors (6, 7). This system enables long-term observation under constant conditions, without the need for feeding, repetitive training, or imposed light–dark schedules.

Transient mechanical perturbations, similar to those occurring in natural encounters or routine handling, serve as simple, nonaversive cues. Here, we examine the postcue reorganization of spontaneous behavior through uninterrupted recordings following a single brief mechanical perturbation, consisting of a gentle aspiration and expulsion using a plastic pipette.

## Results

**Fig. 1.**
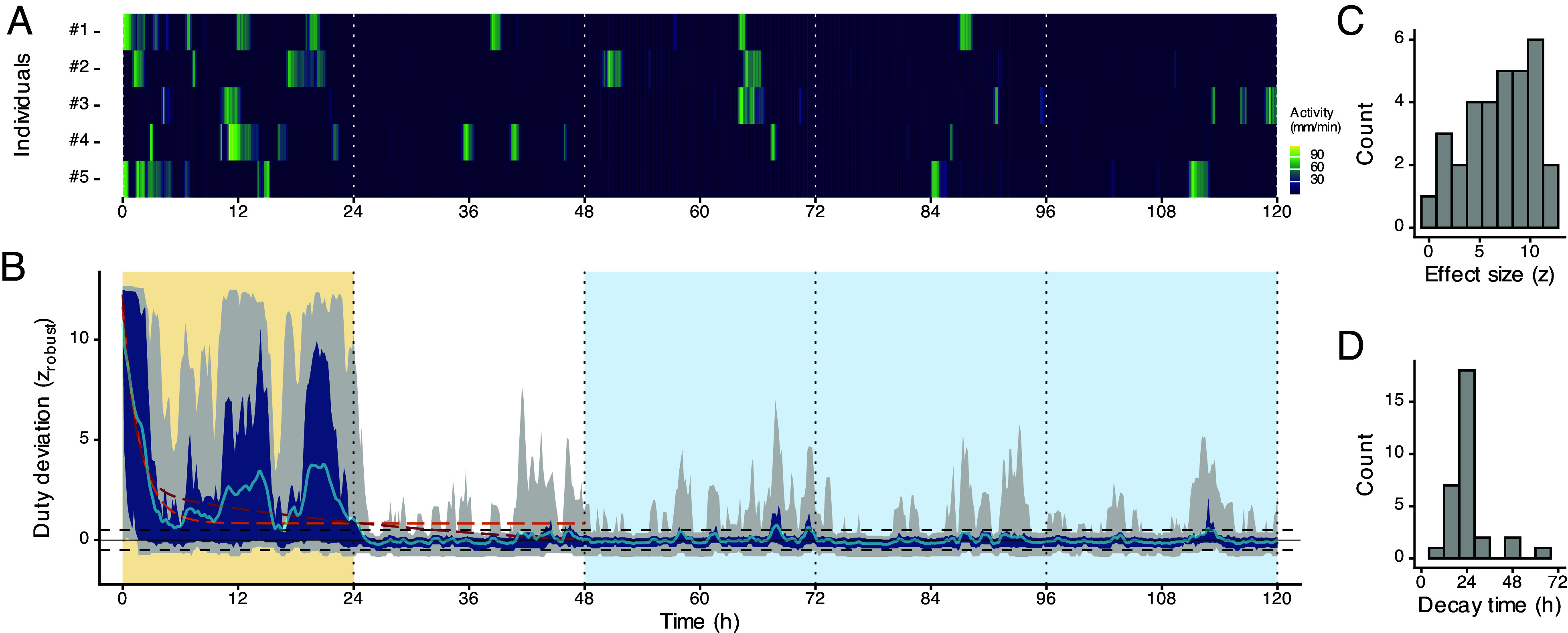
Day-scale carryover in spontaneous rest-activity after a single brief mechanical cue. (*A*) Representative actograms with no additional stimulation after the brief mechanical cue; time zero indicates cue delivery. (*B*) Population summary of duty deviation (robust z-score), shown as the mean (light blue), interquartile range (dark blue), and 10 to 90% range (light gray), with single- and double-exponential fits overlaid as orange and red dashed lines. Shading indicates the early (0 to 24 h) and late (48 to 120 h) windows. The dashed horizontal line indicates late-window reference level (±0.5 z band). (*C* and *D*) Distributions of individual effect sizes and decay times. n = 32.

Representative multiday actograms show that planarians spontaneously alternate between bouts of activity and rest over multiple hours. These bouts exhibit highly irregular timing and spacing, without a consistent 24-h circadian component (Fig. 1*A* and Movie S1). Instead, the observed patterns feature irregular alternations on an ultradian timescale, with activity and rest recurring over several hours (8). Following the cue, active bouts appeared in rapid succession, resulting in a sustained elevation in overall locomotor activity, with the timing of these bout sequences varying across individuals.

To characterize this temporal organization without assuming a specific timescale, we quantified the fraction of time each individual spent in the active state. Following the transient event, the active fraction declined sharply over the first few hours. However, it subsequently increased again and decayed slowly, taking approximately 24 h to approach late-time baseline levels (Fig. 1*B*). This trajectory was more closely approximated by a double-exponential fit than by a single-exponential fit, although neither captured the rebound phase. The initial effect size and decay time were extracted for each individual, revealing that the postcue elevation spans a broad, stochastically distributed temporal window (Fig. 1 *C* and *D*). These results demonstrate that a brief, low-salience exogenous cue induces a day-scale carryover in spontaneous activity.

**Fig. 2.**
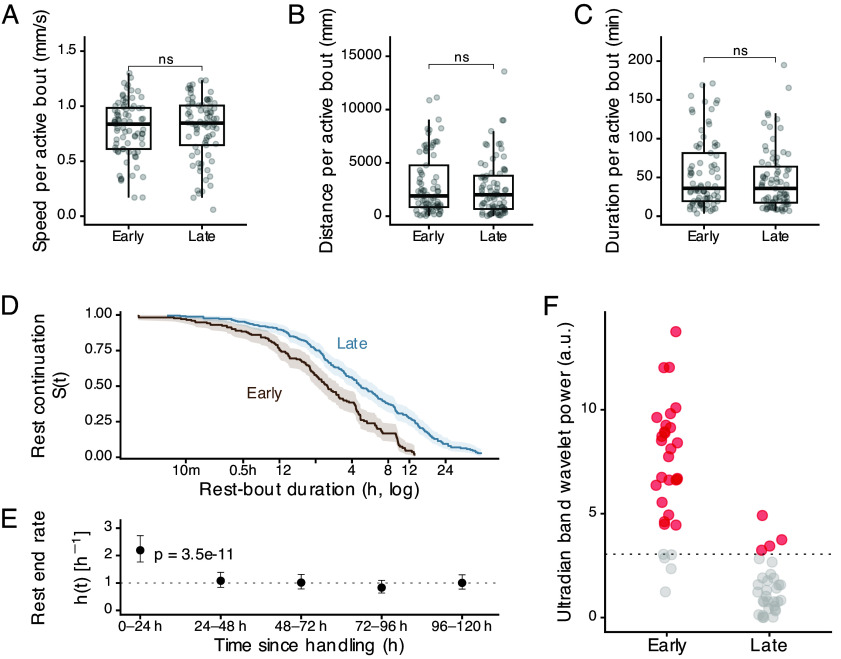
Early activity bias reflects shorter rest bouts with persistent ultradian modulation. (*A*–*C*) Mean active-bout speed, distance, and duration in the early and late windows. (*D*) Rest-bout persistence in early and late windows. (*E*) Rest-ending tendency ratio (early/late) with 95% confidence intervals; values >1 indicate more frequent rest endings in early. (*F*) Ultradian modulation after cue delivery, shown as 2 to 12 h band power and centroid period; dashed line, surrogate threshold. Early, 0 to 24 h; late, 48 to 120 h.

We next investigated whether this elevated activity arose from altered intra-bout motor characteristics. We compared within-bout speed, per-bout path length, and bout duration between the early (0 to 24 h) postcue interval and a late reference window (48 to 120 h). No consistent differences were observed in these kinematics (Fig. 2 *A*–*C*). Instead, the probability of rest-bout continuation was lower and declined more rapidly in the early window, indicating shortened rest-bout durations (Fig. 2*D*). Consistently, the transition probability from rest to activity was significantly higher in the early window (Fig. 2*E*). This demonstrates that shortened rest-bout durations drive a day-scale tonic bias toward activity by redistributing state occupancy, rather than through modification of locomotor kinematics.

Finally, we quantified modulation within the ultradian band corresponding to these several-hour alternations. This temporal patterning was prominent during the early window; however, in the late window, rest bouts lengthened significantly, reducing the frequency of state transitions and leading to a decline in spectral power (Fig. 2*F*). Despite this power decline, the centroid period remained nearly constant. This pattern supports an attenuation of modulation amplitude rather than a shift in characteristic period. These results highlight a two-timescale organization following cue delivery, where an ultradian alternation provides a scaffold for a day-scale elevation in overall activity.

## Discussion

Our findings demonstrate that a single brief event can influence behavior for approximately 24 h. This timeframe defines a persistence window that characterizes the functional reach of the event’s impact. The mechanical cue does not enhance motor capacity per se, but instead lowers the responsiveness threshold for transitions from rest to activity. This targeted shift supports the concept that internal states act as stable biases modulating action selection probability (4).

At each successive transition, a subtle bias toward earlier activity onset is expressed. These minor shifts accumulate into several hours of additional activity as the animal’s behavior gradually returns to a steady state. This accumulation underscores the multiscale organization of behavior, integrating rapid sensorimotor reactions into longer-term internal dynamics (9). The fact that planarians exhibit day-long persistence despite their compact nervous systems suggests that such circuitry is sufficient to support behavioral carryover across a day. The underlying mechanisms may include an early component that increases active-state occupancy soon after cue delivery and a later component sustained by systemic chemical signaling, metabolic shifts, or receptor-mediated pathways with slow kinetics (10–12). This prolonged effect provides empirical insight into the slower regulatory influences that stabilize resting states.

Rather than reflecting an adaptation to stimulus periodicity, this day-scale carryover likely represents a trade-off in the temporal integration of sensory signals (13). It stabilizes state-dependent transitions while potentially delaying reversal when conditions change. A bias that decays over extended windows reduces sensitivity to brief, random perturbations, which prevents frequent state switching and balances the need for behavioral stability with flexibility. In this framework, the 24-h recovery period functions as an integration window, reflecting the natural timescale of environmental constancy. In natural environments, stimuli are multidimensional, and even weak stimuli can produce distinct effects depending on modality and valence. Frequent environmental perturbations cause successive effects to overlap before earlier influences can decay. This overlap leads to interference or shifts in behavioral dimensions, showing that behavior adopts a context-dependent bias shaped by recent history rather than drifting in only one direction. These history-dependent effects persist for at least 1 d and may provide a general framework for interpreting state shifts across behavioral paradigms. Finally, this carryover effect has important practical implications for behavioral assays, which typically require initial handling (14). If the return to homeostasis involves a nonmonotonic trajectory with day-scale carryover, a single initial handling event may confound the entire experimental record. Distinguishing slow background drift from more rapid modulation provides a robust framework for determining when baseline measurements have truly stabilized.

## Materials and Methods

Planarians (*D. japonica*) of the GI clonal line were placed individually in recording chambers under constant darkness. After habituation, each animal received a brief mechanical cue by gentle aspiration into a plastic transfer pipette and immediate release. Locomotor activity was recorded for 120 h without further disturbance by infrared time-lapse imaging at 10-s intervals. Trajectories were extracted with a deep learning-based detector, and the resulting time-series data were analyzed to quantify behavioral state transitions, bout kinematics, and ultradian structure. Detailed methods can be found in *SI Appendix*.

## Supplementary Material

Appendix 01 (PDF)

Movie S1. **Representative single planarian with overlaid detections and tracking.** Playback at 1200x real time. Green bounding boxes indicate per-frame deep-learning detections; the magenta point indicates the tracked position in each frame after post-processing to a single-target track. A qualitative alternation between movement and rest is apparent, and during the initial post-cue period shown here, occupancy of the active state appears denser. The animal shown corresponds to individual #1 in Fig. 1A.

Movie S2. **Procedure for mechanical cue application and close-up images before and after perturbation.** The movie shows the delivery of a single, brief mechanical perturbation, performed by gently drawing the planarian into a plastic transfer pipette and then immediately releasing it. Close-up views obtained immediately before and after the procedure show no obvious macroscopic damage.

## Data Availability

Raw tracking data, analysis code/scripts, trained planarian detector model (weights) data have been deposited in GitHub (https://github.com/Planarian-Brain/daylong-imprint) (15).
