# Supplementary material for: A single brief cue leaves a day-long internal state imprint in planarians
Source: Proc Natl Acad Sci U S A. 2026 Jun 16;123(25):e2606749123. doi: 10.1073/pnas.2606749123 (PMC13291662; doi:10.1073/pnas.2606749123)
Supplement: Supplementary file 1 — Appendix 01 (PDF) [file pnas.2606749123.sapp.pdf]

PNAS

Supporting Information for
A single brief cue leaves a day-long internal state imprint in planarians.

Ojeiru Felix Ezomo, Rena Suzuki, Maria Narahashi, Satoshi Matsuo, Takeshi Inoue

Takeshi Inoue
Email: inoue.t@tottori-u.ac.jp

This PDF file includes:

Supporting text
Legends for Movies S1 and S2
SI References

Other supporting materials for this manuscript include the following:

Movie S1
Movie S2

Supporting Information Text

METHODS DETAILS

Animals and husbandry

The GI clonal line of the freshwater planarian *Dugesia japonica* (1) was maintained at 21 ± 0.5 °C under a 12-h light/12-h dark cycle (lights on 06:00-18:00) with dim white illumination during the light phase (2). Rearing water was tap water dechlorinated with sodium thiosulfate (3). Animals were fed chicken liver at regular intervals and were fasted for 7 days before experiments to minimize variability due to recent feeding and to avoid feeding-associated perturbations during subsequent multi-day recordings. All behavioral assays used individuals measuring 7 mm in total length.

Recording environment and apparatus

Temperature was maintained at 21 ± 1 °C (4), logged every minute, and subsequently confirmed to be stable. Imaging illumination was provided by 850 nm infrared (IR) LEDs. Custom 3D-printed black plastic assay chambers (internal diameter, 9 cm) were prefilled with 40 mL of rearing water (depth ≈ 6.3 mm) and each housed a single planarian. Each animal was left undisturbed in the assay chamber for at least 36 h in constant darkness. It was then given a brief mechanical stimulus by aspirating the animal once into a plastic transfer pipette and releasing it back into the assay chamber (Movie S2). Recording started at time zero (t = 0) immediately after the standardized cue delivery. No feeding, water exchange, or additional stimulation occurred during the 120-h recording in constant darkness. Recordings were initiated in staggered batches at different clock times. After recording, all animals were examined to verify normal morphology and behavior indicative of a healthy state. In an additional validation series performed under the same conditions, the same pipette manipulation was repeated across 50 trials under the present conditions, and no obvious macroscopic damage or overt impairment of posture or locomotion was observed immediately after cue delivery. To confirm that incidental vibrations would not confound behavior, we briefly applied floor vibrations to the apparatus support outside the enclosure; no measurable change in locomotor activity was observed. Time-lapse imaging used a Raspberry Pi Camera Module v3 NoIR mounted above the chamber with the optical axis perpendicular to the water surface, controlled by a Raspberry Pi 4 Model B. Frames were acquired every 10 s (0.1 Hz) at a fixed resolution of 4608 × 2592 pixels, with fixed focus and magnification. To limit evaporation and air currents, the camera and chamber were enclosed in a transparent cylindrical shroud with a clear lid, forming a near-sealed enclosure. Recordings were excluded if remaining water volume was < 35 mL at the end of the session or if the animal underwent asexual fission during recording.

Tracking and validation

Images were annotated for training using the open-source tool LabelImg (5). For object detection and centroid extraction, each frame was analyzed with a custom planarian detector based on YOLO11 (Ultralytics) (6), trained on images annotated under the present experimental conditions. The centroid of each predicted bounding box was taken as the animal's position. Because one animal was present per chamber, cross-frame data association was unnecessary. To address tracking dropout, occasional missed detections due to surface reflections or IR glare were linearly interpolated in x and y between the nearest valid positions on the native 10 s time base. In the same routine, false positives were removed using an inter-frame displacement cap and a confidence threshold. For each individual, trajectories were visually verified by overlaying centroid tracks on raw frames to confirm correspondence with actual motion (Movie S1).

Quantification and statistical analysis

Time-series preprocessing.

For each individual, frame-to-frame displacement magnitudes were aggregated into non-overlapping 1-min intervals. To reduce high-frequency noise while preserving temporal resolution, these 1-min intervals were smoothed with a 10-min rolling average calculated at 1-min steps. These smoothed time series were truncated to 0-120 h and used for subsequent analyses and visualization.

Population-level duty analysis.
The smoothed locomotor signal was log-transformed and partitioned into low- and high-activity modes by two-cluster k-means. The default decision threshold was placed at 0.40 of the distance from the low-activity to the high-activity centroid on the log scale, with symmetric hysteresis. Minimum bout durations were enforced by relabeling active runs < 30 s as rest and rest runs < 60 s as active. To assess the robustness of state segmentation, the full analysis was repeated after shifting the threshold weight from 0.40 to 0.30 or 0.50 (±25%) and after tightening or relaxing the minimum bout criteria to 60/120 s or 15/30 s for active/rest bouts, respectively. Duty, the fraction of time spent in the active state, was computed over 60-min trailing windows, updated each minute, and indexed to the window end. Two non-overlapping analysis windows were defined to separate estimation of early post-cue deviation (Early, 0-24 h) from estimation of a stable reference segment (Late, 48-120 h) used for standardization and window-based contrasts. The Late window was set apart from the immediate post-cue period to support robust individual-wise centering and surrogate calibration in ultradian analyses. For a common scale, duty was centered at each individual's Late window mean and scaled by the median absolute deviation, yielding a robust z-score series. Population summaries pooled standardized values in 10-min bins using trimmed means and quantiles. To examine whether the post-cue recovery was better captured by one or two characteristic timescales, the population trajectory was also compared with single- and double-exponential fits over the post-cue period, and descriptive fit quality was compared by AIC.

Inter-individual variability
To summarize inter-individual variability in behavioral responses, two individual-level measures were calculated from each planarian's duty-deviation time series: effect size, as a measure of response magnitude, and decay time, as a descriptive measure of persistence duration. To extract macroscopic state transitions while reducing the influence of short-lived ultradian activity bursts and transient rest periods, the 10-min binned individual z-score time series was smoothed with a 4-h centered moving average. Effect size was defined as the maximum smoothed z-score during the first 12 h after cue delivery. Decay time was assessed across the 120-h post-cue analysis window and was defined as the earliest time point at which an individual's smoothed z-score fell below the 0.5 reference threshold and remained below this threshold for at least 12 h.

Active-bout construction and kinematic measures.
Consecutive samples sharing the same inferred state were merged into runs and summarized within each analysis window (Early and Late). This separation, as described above, supports robust individual-wise centering and surrogate calibration in ultradian analyses by isolating the stable reference segment (Late window) from the initial post-cue period. Because runs could extend across a window boundary, bouts crossing the start or end of a window were truncated at that boundary, and the within-window segment only was used to compute bout-level measures; such bouts were marked as boundary-censored. Bout duration was computed as the sum of inter-sample intervals within the window, and bout distance was computed by integrating instantaneous speed over these intervals. Mean speed per bout was defined as bout distance divided by bout duration. Only active bouts were retained for downstream comparisons. Distributions of per-bout speed, distance, and duration were compared between Early and Late windows using two-sided two-sample t-tests.

Rest-bout construction and mixed-effects survival modeling.
Rest persistence, defined as the probability that a rest remains ongoing over time, and the rest-end rate, defined as the instantaneous rate at which rests terminate, were quantified. Consecutive samples in the same state were merged into bouts; rest-bout durations were measured in hours within the Early (0-24 h) and Late (48-120 h) windows, and bouts crossing a window boundary were treated as right-censored at that boundary. Survival analysis was used because rest-bout durations were heavy-tailed and frequently right-censored by the window limits, and because it allows comparison of rest-end rates between windows while accounting for repeated bouts within individuals. Duration distributions were summarized with Kaplan-Meier curves on a logarithmic time axis with 95% confidence bands. For inference, we fit a mixed-effects Cox proportional hazards model with window (Early vs Late) as a fixed effect and

individual identity as a random intercept, estimating the rest-end rate in Early relative to Late; P values were obtained from Wald tests.

Trend-phasic decomposition for slow drift and ultradian structure.
Ultradian organization was quantified from the phasic duty component using a Morlet continuous wavelet transform with fixed sampling step and scale spacing. Band-integrated power within 2-12 h and the power-weighted centroid period were computed by averaging power over time within each analysis window and integrating across period with the trapezoid rule. To control for apparent band energy from slow drift or short-range dependence, 1 h block-shuffle surrogates, n = 200 per individual, defined a 95th-percentile null threshold from the Late window. Early-Late contrasts were interpreted relative to this null.

Implementation.
Analyses were performed in R, using standard packages for data manipulation, time-series analysis, and survival modeling.

Declaration of generative AI and AI-assisted technologies
The authors acknowledge the use of ChatGPT for language editing support, as well as for code generation and debugging. The authors reviewed and validated all outputs generated with these tools and take full responsibility for the content of the publication.

Data, Materials, and Software Availability
The data, code, and custom planarian detector model are publicly available on GitHub at https://github.com/Planarian-Brain/daylong-imprint.

Figures

Movie S1 (separate file). Representative single planarian with overlaid detections and tracking.
Playback at 1200× real time. Green bounding boxes indicate per-frame deep-learning detections; the magenta point indicates the tracked position in each frame after post-processing to a single-target track. A qualitative alternation between movement and rest is apparent, and during the initial post-cue period shown here, occupancy of the active state appears denser. The animal shown corresponds to individual #1 in Fig. 1A.

Movie S2 (separate file). Procedure for mechanical cue application and close-up images before and after perturbation.
The movie shows the delivery of a single, brief mechanical perturbation, performed by gently drawing the planarian into a plastic transfer pipette and then immediately releasing it. Close-up views obtained immediately before and after the procedure show no obvious macroscopic damage.

SI References

1. H. Orii, K. Agata, K. Watanabe, POU-domain genes in planarian *Dugesia japonica*: the structure and expression. *Biochem Biophys Res Commun* **192**, 1395-1402 (1993).
2. T. Inoue, K. Agata, Quantification of planarian behaviors. *Dev. Growth Differ.* **64**, 16-37 (2022).
3. M. Mori *et al.*, Calcium ions in the aquatic environment drive planarians to food. *Zoological Lett.* **5**, 31 (2019).
4. T. Inoue, T. Yamashita, K. Agata, Thermosensory signaling by TRPM is processed by brain serotonergic neurons to produce planarian thermotaxis. *J. Neurosci.* **34**, 15701-15714 (2014).
5. Tzutalin (2015) LabelImg: Graphical image annotation tool. Git code (HumanSignal, GitHub).
6. G. Jocher, J. Qiu (2025) Ultralytics YOLO11 Documentation.
